# Synchronous Acute Appendicitis and Acute Cholecystitis

**DOI:** 10.7759/cureus.37248

**Published:** 2023-04-07

**Authors:** Luis F Flores, Álvaro Morillo Cox, Tatiana Fernandez Trokhimtchouk, Estefanie S Otañez, Andrés V Ayala

**Affiliations:** 1 General Surgery, Universidad Internacional del Ecuador/Axxis Hospital, Quito, ECU

**Keywords:** surgical acute abdomen, laparoscopy, synchronous, appendicitis, cholecystitis

## Abstract

The occurrence of synchronous acute cholecystitis and appendicitis is rare. There are few cases reported and small series in the literature. We report the case of a 77-year-old male who presented to the emergency department with right-sided abdominal pain. He was diagnosed preoperatively with acute calculous cholecystitis. During the initial laparoscopy, a complicated appendiceal phlegmon was found and was treated with a one-step laparoscopic approach and subsequent antibiotics. The patient had an uneventful recovery and was discharged on postoperative day (POD) 2. The pathology report confirmed both diagnoses and an incidental low-grade appendiceal mucinous neoplasm. Although uncommon, it is important to be aware of the possibility of both simultaneous pathologies in a patient who presents to the emergency department with abdominal pain.

## Introduction

Acute appendicitis is the most common diagnosis for emergency abdominal surgery worldwide [1]. Meanwhile, acute cholecystitis due to gallstone disease is yet another common cause of acute abdominal pain, which requires urgent surgery [2]. Both are among the most common pathologies in general surgical practice, although their synchronous presentation in a single patient is rare. It is important to consider the possibility of this double diagnosis, especially in patients who present with acute abdominal pain that is ambiguous, and when the anamnesis and physical examination suggest a mixed picture [3].

We aim to report the incidental finding of complicated acute appendicitis during laparoscopic cholecystectomy for acute calculous cholecystitis in an elderly patient. This highlights the importance of a proper presurgical evaluation and of laparoscopic exploration of the entire abdominal cavity in all patients presenting with surgical acute abdomen, as various pathologies may coexist.

## Case presentation

A 77-year-old male, with no relevant medical history, presented to the emergency department of our hospital with a history of 12-hour intense, non-radiating right upper quadrant pain of sudden onset. The pain was described as sharp, and it was triggered by copious food ingestion; no nausea or vomiting was reported. He denied jaundice, fever, changes in bowel movements, or urinary symptoms.

He reported having visited another healthcare facility 10 days ago complaining of diffuse abdominal pain associated with nausea, vomiting, and diarrhea. Upper abdominal ultrasonography was done at that time with no significant findings, and blood tests were within normal limits. He was diagnosed with inflammatory enterocolitis and was referred to a gastroenterologist, who treated the episode symptomatically with oral rehydration, a proton pump inhibitor, and probiotics. The symptoms resolved completely within two days.

Upon new admission, he had normal vital signs, no fever, and no tachycardia. Abdominal examination was remarkable for distension, diminished bowel sounds, and tenderness on light palpation on the right hemiabdomen with local guarding. Pain limited the exploration of signs of peritoneal irritation, and there were no palpable masses.

**Figure 1:**
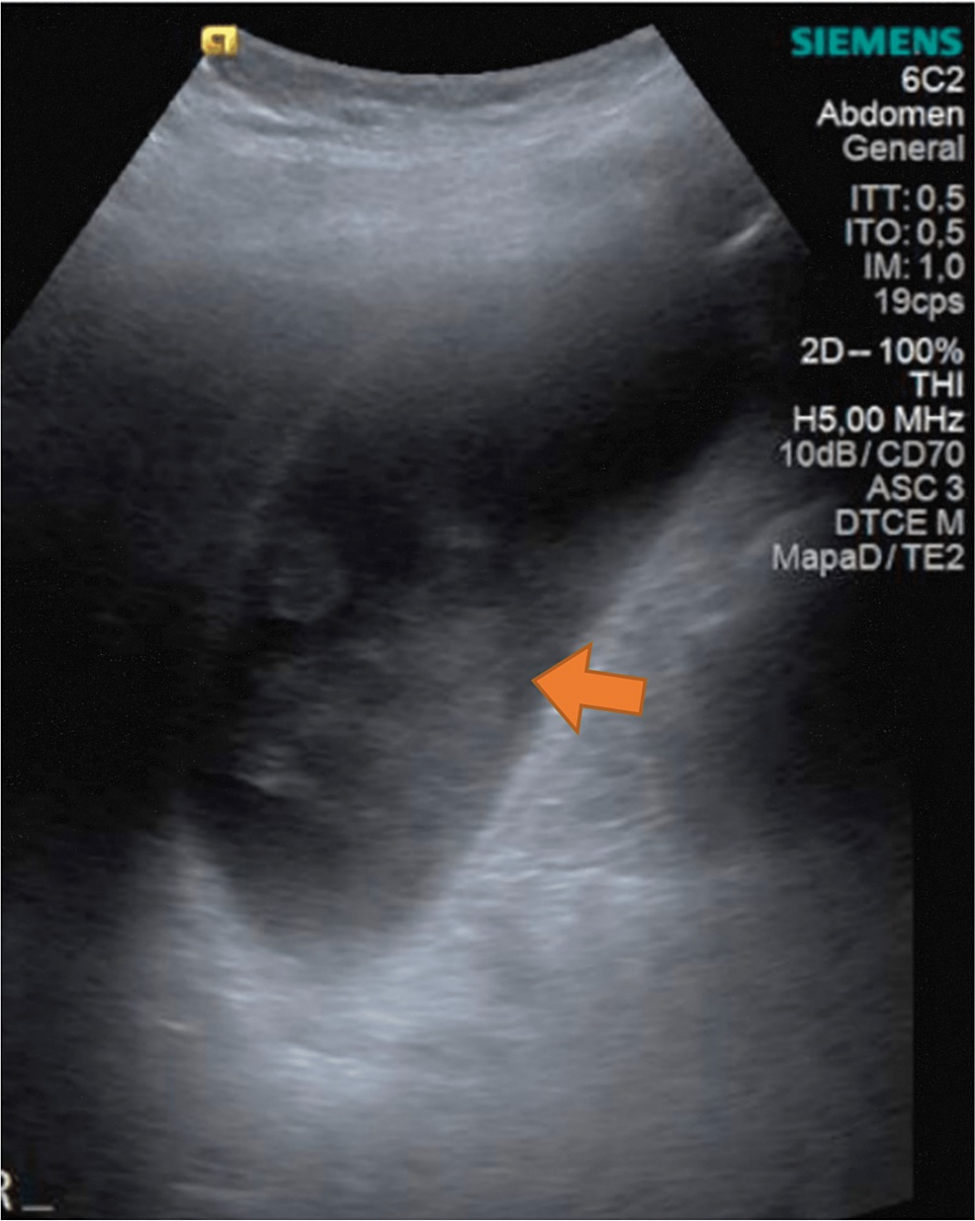
Gallbladder ultrasound showing a markedly dilated gallbladder with multiple small clustered images (orange arrow) suggesting biliary sludge

The initial blood tests reported a white blood cell count of 13,040/mm^3^ (normal range: 4,320-10,421/mm^3^) with 84% (normal range: 50%-70%) neutrophils, a C-reactive protein of 60.1 mg/L (normal range: 0-5 mg/L), and normal liver function tests. A new upper abdominal ultrasound was ordered, which reported a markedly dilated gallbladder with a volume of 116 mL, a 5-mm wall with diffuse thickening, and hyperechogenic multiple small clustered images inside suggesting biliary sludge. The common bile duct diameter was within the normal range. There was no free peritoneal fluid present (Figure 1).

Intravenous fluids were initiated, along with antibiotics (ampicillin + sulbactam 3 g IV) and analgesia. He was admitted with a diagnosis of grade I (mild) cholecystitis applying the Tokyo 2018 criteria [4] and planned for laparoscopic cholecystectomy. No contraindications were present for the procedure.

**Figure 2:**
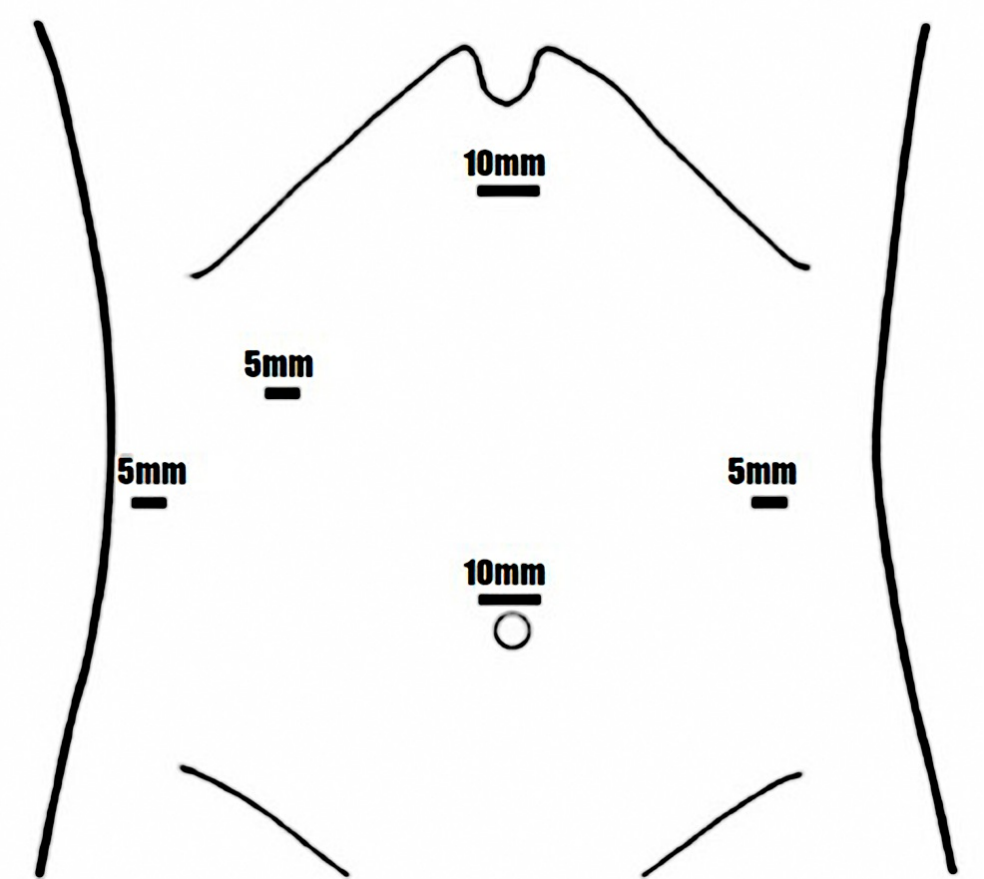
Trocar placement

The surgical approach began with an umbilical incision, and an open technique was used for pneumoperitoneum creation. During the initial laparoscopy, an inflammatory phlegmon involving the greater omentum, cecum, terminal ileum, gallbladder, and appendix was identified. Scarce free peritoneal fluid was found in the right paracolic gutter down to the pelvis. Additional ports were placed under laparoscopic guidance: a 10-mm subxiphoid port, two right upper quadrant 5-mm ports, and a left flank 5-mm port (Figure 2).

**Figure 3:**
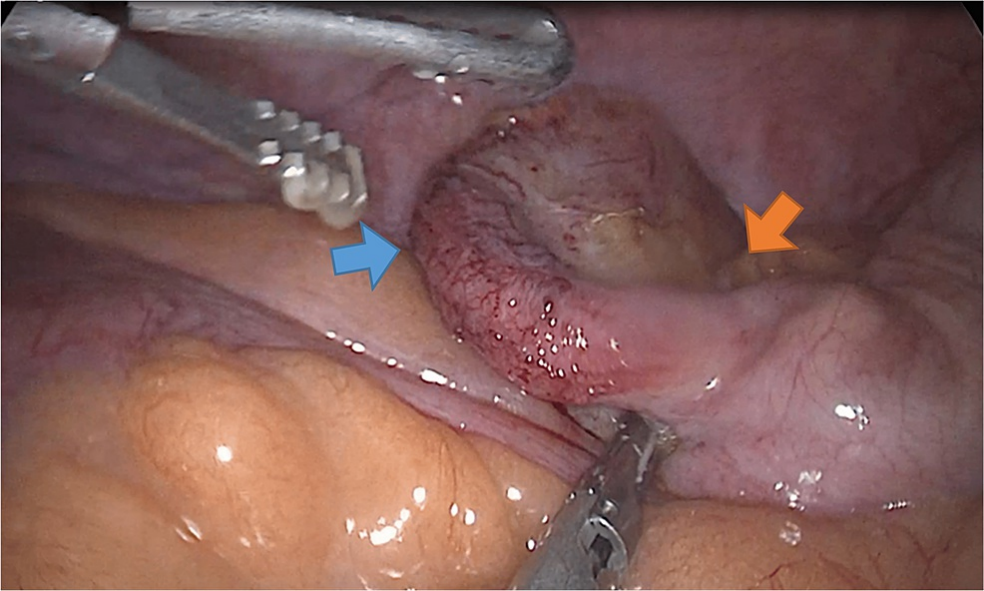
Inflamed appendix with areas of necrosis (blue arrow); orange arrow points at the appendicular base

**Figure 4:**
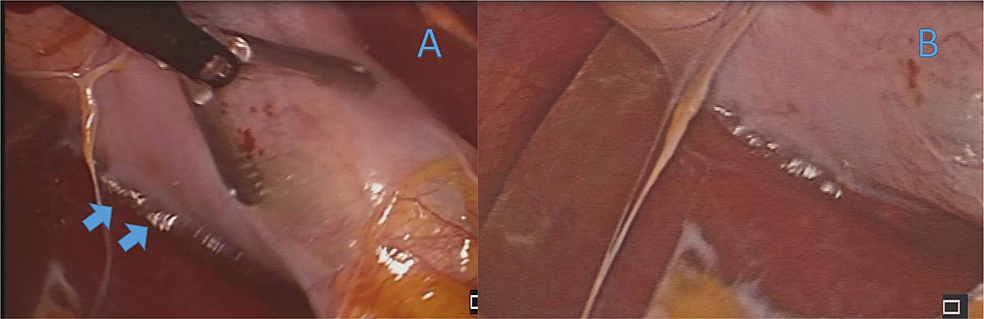
(A) Gallbladder with signs of inflammation: thickened and tense edematous wall (blue arrows); (B) close-up view

The appendix was difficult to locate because of adhesions and medial rotation of the cecum with the appendiceal tip pointing anterosuperiorly. Necrosis and perforation were identified in the middle third of the vermiform appendix (Figure 3). The gallbladder was found to be severely inflamed and distended, with an edematous and thickened wall, along with gross omental adhesions (Figure 4). There were no other relevant findings in the abdominal cavity. An appendectomy was performed, followed by a cholecystectomy. A closed-suction drain was left in place extending from the gallbladder bed area down to the pelvis along the right paracolic gutter.

The postoperative course was uneventful. Liquid oral intake was reinitiated 12 hours after the surgery and was well tolerated. The patient tolerated oral intake on his postoperative day (POD) 1. The drain output was low and serosanguinous. The patient reported passing flatus on POD 1. The patient was discharged on POD 2 after drain removal. Oral antibiotics were prescribed for five more days. The histopathology report confirmed acute cholecystitis (with microlithiasis) and necrotic acute appendicitis plus an incidental finding of a low-grade mucinous neoplasm on the distal third (pTis).

## Discussion

Finding acute cholecystitis simultaneously with appendicitis is a rare condition, although individually both are very common acute conditions in the emergency department. In the USA, gallbladder disease affects approximately 20 million individuals yearly, and more than 200,000 people are diagnosed with acute cholecystitis each year [2]. On the other hand, the global annual incidence of appendicitis is 96.5-100 cases per 100,000 people in the adult population [1].

According to the literature, patients with those two simultaneous conditions are difficult to evaluate in the emergency room. They generally present with right upper quadrant pain and right-sided or diffuse abdominal tenderness, so it can be challenging to reach a high level of suspicion based on the clinical picture alone [3,5]. Each pathology has characteristic findings on the physical examination; for example, McBurney’s, Rovsing’s, and Blumberg’s signs are regularly present in acute appendicitis as Murphy’s sign is in acute cholecystitis. However, their absence does not exclude the diagnosis, especially when pain limits eliciting these signs as in our patient [3,6].

Upper abdominal ultrasound is the standard of care when gallbladder pathology is suspected, while contrast-enhanced computed tomography (CT) has become the choice to evaluate lower abdominal pain. Abdominal CT scan has led to reduce false negatives for appendicitis and decrease misdiagnoses. In terms of cholecystitis, a sonogram is preferred over CT as approximately 8% of gallstones are isodense to the surrounding bile [3]. Still, ultrasonography is operator-dependent. In our case, only an ultrasound test was done as we missed suspicion of appendicitis. However, with the initial diagnosis, he had an indication for urgent surgical management [6].

Alkhurmudi et al. [5], in a previous report, discussed two theories that can explain the occurrence of this synchronous presentation. One consists of secondary appendicitis that can develop in conjunction with other intra-abdominal inflammatory pathologies when the appendix lies near the site of inflammation. The other refers to the possible occurrence of acute cholecystitis secondary to appendicitis as the result of direct invasion or translocation of bacteria from the muscularis propria of a gangrenous appendix into the portal venous system. The latter can produce contamination of the bile, which finally causes gallbladder inflammation.

In a retrospective analysis, it is relevant to consider that our patient presented an episode of abdominal pain 10 days before the latest admission that was treated as inflammatory enterocolitis and had full resolution of symptoms. At that time, no apparent abnormalities were found in complementary studies. We suspect that this previous episode could be the initial manifestation of acute appendiceal inflammation, which then led to complicated appendicitis and contiguous inflammation of the gallbladder. Nonetheless, we cannot rule out the biliary sludge (microlithiasis) as the culprit of concomitant cholecystitis [2,7].

Histopathologic and trans-operatory findings are consistent with our hypothesis as the most compromised organ was the appendix. While necrosis was present in the appendix specimen, the gallbladder only showed mild acute cholecystitis without signs of gangrene. The incidental finding of a low-grade mucinous neoplasm (pTis) at the tip of the appendix does not influence the management, and their implications are beyond the discussion of this report [8].

Finally, regarding the surgical approach, previous studies have demonstrated that a one-step laparoscopic procedure is feasible and safe for treating both illnesses [9]. Our patient underwent a single intervention with a favorable outcome as described above.

## Conclusions

Among the most common causes of abdominal pain that require surgical intervention are acute appendicitis and acute cholecystitis. Although a rare occurrence, it is important to consider the possibility of both occurring simultaneously in a patient presenting with nonspecific abdominal pain to the emergency department. The condition can be safely solved by a simultaneous laparoscopic appendectomy and cholecystectomy without delay to avoid complications such as intra-abdominal sepsis. Surgeons must be aware of this circumstance so that preoperative workup can be successfully done and optimal treatment planned. It is imperative that a thorough exploration of the abdominal cavity be always performed as the first step of laparoscopic surgery.
